# Ventilator-associated pneumonia in a trauma ICU

**DOI:** 10.1186/cc14399

**Published:** 2015-03-16

**Authors:** M Raja, A Ely, P Zolfaghari

**Affiliations:** 1 Royal London Hospital, London, UK

## Introduction

Ventilator-associated pneumonia (VAP) is associated with increased length of ventilation, ICU stay, mortality, cost and antibiotic burden [1]. There is a large variation in reported rates of VAP, partly as a result of inconsistencies in definition [2]. We explored a more pragmatic definition to describe the VAP rate, antibiotic burden and outcome of VAP in a 44-bed adult critical care unit in a level 1 trauma centre.

## Methods

A retrospective review of all adult patients admitted to the ICU at The Royal London Hospital over a 6-month period (February to August 2014). The diagnosis of VAP was based on the Clinical Pulmonary Infection Score. Patients were identified with VAP if they were started on antibiotics for chest sepsis 48 hours after start of mechanical ventilation. Demographic, clinical, microbiological and radiological data were collected to identify risk factors, and compare VAP and non-VAP groups. Chi-squared and ANOVA tests were performed using the SOFA statistics package.

## Results

A total of 535 mechanically ventilated patients were admitted in the study period, with 281 ventilated for more than 48 hours. The incidence of VAP was 11% in all ventilated patients and 19.6% in those ventilated more than 48 hours. VAP rates were 31% in polytrauma, 25% in neurotrauma and 18% in the neuromedical/surgical cohort. Early and late onset VAP were equal in number. Patients with VAP spent longer on mechanical ventilation (9 ± 9 in no VAP vs. 18 ± 18 days in VAP patients; *P *< 0.001), and had longer ICU and hospital LOS (11 ± 10 vs. 22 ± 20 days; *P *< 0.001). However, APACHE II scores and hospital mortality were unaffected (VAP 33.3% vs. no VAP 37.6%; *P *= 0.173). Despite rising inflammatory markers and secretion load, many patients did not exhibit oxygenation deficits. Sputum microbiology showed *S. aureus*, *H. Influenza*, Klebsiella and Enterobacter as predominant pathogens with low rates of Pseudomonas, Acinectobacter and other resistant organisms. Average length of antibiotic use was 6 (3 to 18) days.

## Conclusion

Chest sepsis after 48 hours of mechanical ventilation commonly complicates neurocritical illness and polytrauma requiring significant ICU resources and antibiotic burden. However, it does not affect mortality. Further research should focus on pathophysiology and new preventative measures to reduce VAP in the at-risk population.
